# Pediatric inflammatory bowel disease in New Zealand study: a 10-year update on national incidence and point prevalence in 2025

**DOI:** 10.1093/crocol/otag117

**Published:** 2026-10-28

**Authors:** Canada T L Cherry, Helen M Evans, Stephen W Mouat, Jonathan R Bishop, Amin J Roberts, Nir Fireman, Ashwin Bhana, Jillian J Haszard, Andrew S Day, Robert N Lopez

**Affiliations:** Department of Paediatric Gastroenterology, Starship Hospital, Starship Child Health, Auckland, New Zealand; Department of Paediatric Gastroenterology, Starship Hospital, Starship Child Health, Auckland, New Zealand; Department of Paediatric Gastroenterology, Starship Hospital, Starship Child Health, Auckland, New Zealand; Department of Paediatric Gastroenterology, Starship Hospital, Starship Child Health, Auckland, New Zealand; Department of Paediatric Gastroenterology, Starship Hospital, Starship Child Health, Auckland, New Zealand; Paediatric Gastroenterology, Tiny Giants Paediatric Specialists, Auckland, New Zealand; Department of Paediatrics, Capital, Coast and Hutt Valley, New Zealand; Department of Paediatric Gastroenterology, Starship Hospital, Starship Child Health, Auckland, New Zealand; Department of Paediatrics, Christchurch Hospital, Christchurch, New Zealand; Department of Paediatric Gastroenterology, Starship Hospital, Starship Child Health, Auckland, New Zealand

**Keywords:** epidemiology, pediatrics, IBD

## Abstract

**Background and Aims:**

The incidence of inflammatory bowel disease (IBD) in children is increasing worldwide, with high rates previously noted in New Zealand. The incidence and prevalence of pediatric IBD in New Zealand was last delineated in 2015. The aim of this study was to provide updated epidemiological data by delineating the incidence and prevalence of pediatric IBD in New Zealand ten years after the previous assessment.

**Methods:**

For the incidence cohort, patients younger than 16 years with IBD, diagnosed between January 1, 2025 and December 31, 2025 were identified and information entered into a secure database. Population data were obtained and incidence calculated. To derive the point prevalence, all patients diagnosed with IBD under the age of 16 years on June 30, 2025 were identified and information entered into a secure database. Population data were obtained and prevalence calculated.

**Results:**

The incidence of pediatric IBD, Crohn’s disease (CD), ulcerative colitis (UC) and inflammatory bowel disease-unclassified (IBD-U) in New Zealand in 2025 was 8.0, 5.6, 2.0, and 0.4 per 100 000 respectively. The point prevalence of IBD, CD, UC, and IBD-U in children in New Zealand on June 30, 2025 was 33.0, 21.7, 8.1, and 3.2 per 100 000 respectively.

**Conclusions:**

The incidence and prevalence of IBD have increased 1.5 times in children in New Zealand since 2015. New Zealand continues to have some of the highest rates of pediatric IBD in the world. Ongoing research is needed to understand factors leading to increasing rates and to ensure appropriate health service provision.

Key Messages

- **What is already known?** 
  The incidence of pediatric inflammatory bowel disease (IBD) is increasing worldwide. The incidence and prevalence in New Zealand were last delineated in 2015.
- **What is new here?** 
  The incidence of pediatric IBD in New Zealand has increased by 53% since 2015 and the prevalence has increased by 52% since 2015.
- **How can this study help patient care?** 
  Due to this increase, it is hoped that the results of this study will help inform optimal healthcare provision for children with IBD across New Zealand.

## Introduction

Inflammatory bowel disease (IBD), which encompasses Crohn’s disease (CD), ulcerative colitis (UC) and IBD-unclassified (IBD-U), primarily affects the gastrointestinal tract and is characterized by episodes of intestinal inflammation.^1^ Although IBD can be diagnosed at any age, up to 20% of cases have their onset in childhood.^2^

Rates of IBD in children have been noted to be increasing worldwide, with particularly high incidence reported in New Zealand.^3^^,^^4^ Furthermore, a recent systematic review based on research between 1950 and 2020 found that the pooled estimate of the annual incidence of IBD in children in Australasia was 4.1 per 100 000 person-years, with the highest incidence rate being 12.8 per 100 000 person-years in Canterbury, New Zealand in 2014.^5^ This report also calculated a pooled prevalence rate in Australasia of 36.0 per 100 000 persons.^5^

IBD has been described as having 4 epidemiological stages; Stage 1 (emergence), with low incidence and prevalence; Stage 2 (an acceleration in incidence), with rapidly increasing incidence and low prevalence; Stage 3 (compounding prevalence), where the incidence decelerates, plateaus or declines and the prevalence increases; and Stage 4 (prevalence equilibrium) whereby the prevalence plateaus.^6^ Recent reports in adults have shown a number of countries in the Western world, including New Zealand, are now in Stage 3 characterized by plateauing of incidence rates.^7^ By contrast, the incidence of IBD in children is continuing to increase in most countries, in keeping with Stages 1 or 2 of this model.^3^

The incidence and prevalence of pediatric IBD in New Zealand was last delineated in 2015, as part of the Pediatric IBD in New Zealand (PINZ) study.^8^^,^^9^ At that time, the incidence and prevalence in children aged less than 16 years old were 5.2/100 000 person-years and 21.7/100 000 persons, respectively.^8^^,^^9^

The underlying hypothesis of the current study was that the incidence and prevalence of IBD in children in New Zealand will have increased since 2015.

The specific aim of this study was to provide updated epidemiological data by delineating the incidence and prevalence of IBD in children in New Zealand in 2025, 10 years after this was last completed.

## Methods

### Ethics and approvals

Approval for the PINZ study was obtained from the Health and Disability Ethics Committee (HDEC) of the Ministry of Health, New Zealand. (HDEC Ethics reference: 2024 EXP 21876).

Locality approval was also obtained from Auckland, Capital, Coast and Hutt Valley and Canterbury districts. In accordance with local research customs Māori consultation was sought, and approval gained, from all 3 districts prior to data collection commencing.

### Healthcare in New Zealand

New Zealand has a public healthcare system which provides specialist level care without charge to residents. In parallel, specialist level care may be accessed in the private sector, by choice and at cost to the consumer.

All patients with IBD in New Zealand are referred from general pediatricians or general practitioners to pediatric gastroenterologists under whose care they remain throughout childhood. Generally, children in New Zealand remain under pediatric care until they are 15 or 16 years old. Pediatric gastroenterologists in New Zealand deal with all aspects of gastroenterology and none sub-specialize solely in IBD. There is 1 quaternary, and 2 tertiary centers who care for children with IBD; Auckland, and Wellington and Christchurch, respectively. All children in New Zealand with IBD have oversight of care by one of these centers. All the pediatric gastroenterologists in New Zealand were involved in the present study. Adult gastroenterologists across New Zealand, including those who work in private practice, were also contacted via the NZ society of gastroenterology, well-established pediatric adult links and by contacting the major private gastroenterology practices in the country, to enter data for any patient who met the study inclusion criteria.

Given the involvement of all pediatric gastroenterologists in the country, the centralization of healthcare within New Zealand and the contact made with key adult gastroenterologists, it is unlikely that there would be many, if any, children who met the study criteria but were not captured by the study.

### Study population

#### Incidence cohort

The inclusion criteria for the incidence cohort were all children aged less than 16 years, diagnosed with IBD anywhere in New Zealand (according to standard criteria)^10^ between January 1, 2025 and December 31, 2025, and who were resident in New Zealand at the time of diagnosis. The diagnosis of IBD was categorized into CD, UC, and IBD-U. Residency was defined as citizenship or permanent residency. This status is required knowledge for healthcare provision in New Zealand since residents are eligible for funded healthcare.

Data collected included sex, age at diagnosis, ethnicity, hospital district and region the patient lived in at diagnosis,^11^ disease phenotype and location, extra-intestinal manifestations and duration of symptoms prior to diagnosis. Data were collected and entered prospectively by the treating gastroenterologist when a patient was diagnosed. Reminders were sent monthly, and again at the end of the study period, to submit data.

#### Prevalence cohort

The inclusion criteria for the prevalence cohort included children aged less than 16 years, normally resident in New Zealand and with a diagnosis of IBD, on June 30, 2025.

Data collected included age on June 30, 2025, sex, ethnicity, hospital district and region the patient resided within,^11^ disease phenotype, and factors reflective of more severe disease (escalation to advanced therapy, requirement for IBD-related surgery and number of hospital admissions since diagnosis). Where diagnosis of IBD changed, the current IBD diagnosis was recorded.

Data were entered by the pediatric gastroenterologists across New Zealand, a research nurse and one of the authors (CTLC) based on clinical records of all patients diagnosed with IBD.

### Data collection

Data were collected from healthcare databases across New Zealand and entered into a secure research electronic data capture (REDCap) database. The REDCap database was maintained by 3 of the authors (C.T.L.C., J.H., and R.N.L.) with ability for all gastroenterologists in the country to input data, either themselves or via an assigned surrogate, in a secure and confidential manner.

In New Zealand, all children under 5 years of age who are diagnosed with IBD get a genetic panel. Those children with a genetic/immunologic component were excluded from the study.

Ethnicity data was recorded as European (Caucasian), Māori (indigenous people of New Zealand), Pacific peoples, Asian, Indian, Middle Eastern/Latin American/African and other ethnicity. Ethnicity reporting was based on that recorded in health databases. These data reflect the ethnic group or groups that a parent identifies with (noting that some children will belong to more than 1 group).

Date of diagnosis was recorded as date of endoscopy, if available, or date of first clinic appointment with gastroenterology after endoscopy.

Admissions since diagnosis were only recorded if they were directly related to the child’s IBD. Admissions to day stay units for iron infusions, biologic administration or endoscopic procedures were excluded.

Extra-intestinal manifestations were recorded as rheumatological, ophthalmological, dermatological, hepatobiliary or other with the option to specify the extra-intestinal manifestation.

### Statistics

The last New Zealand census was undertaken in 2023 by Stats NZ.^12^ A census is held every 5 years and for years in between, population estimates are calculated.^12^ The estimated population from 0 to 15 years on June 30, 2025 in New Zealand was obtained from Stats NZ^13^ along with population figures for 0-15 years by geographic region.

The incidence of pediatric IBD in New Zealand between 1 January and December 31, 2025 was calculated for IBD, CD, UC, and IBD-U with the number of new diagnoses acting as the numerator and the population estimate for that year acting as the denominator. This is reported per 100 000 person-years.

The point prevalence of pediatric IBD, CD, UC, and IBD-U in New Zealand on June 30, 2025 was calculated with the number of existing diagnoses serving as the numerator and the population estimate for 2025 serving as the denominator. This is reported per 100 000 persons. Since these are population estimates, confidence intervals are not reported.

The 2 cohorts are described in terms of age at diagnosis (median and interquartile range [IQR]), sex, and by region. A map of regional prevalence was generated. Extra-intestinal manifestations at diagnosis and treatments are described using descriptive statistics. All available data are described. All analysis was carried out in Stata 18.0 (StataCorp, Texas).

STROBE (Strengthening the Reporting of Observational Studies in Epidemiology) criteria were used when reporting this data.

## Results

### Incidence cohort

#### Incidence of pediatric IBD in New Zealand in 2025

The number of children diagnosed with IBD in New Zealand between January 1, 2025 and December 31, 2025 aged between 0 and 15 years and 364 days was 83. There were 58 (69.9%) diagnosed with CD, 21 (25.3%) diagnosed with UC and 4 (4.8%) diagnosed with IBD-U (Table 1). The ratio of CD: UC was 2.7:1.

**Table 1:** Incidence, age, and sex of newly diagnosed IBD in children aged 0-15 years inclusive in New Zealand in 2025 and by IBD subtype (*n* = 83) including incidence in 2015.

Characteristic	IBD	CD	UC	IBD-U
***n***	83	58	21	4
**Incidence in 2025, per 100** **000 person-years^a^**	7.96	5.56	2.01	0.38
**Incidence in 2015, *n* (incidence per 100** **000 person-years)**	51 (5.2)	34 (3.5)	10 (1)	7 (0.7)
**Age, median (IQR) years**	12.0 (10, 14)	12.2 (10, 14)	12.8 (11, 14)	7.5 (5.6, 11.5)
**Sex, *n* (%)**				
** Male**	53 (63.9)	40 (69.0)	12 (57.1)	1 (25.0)
** Female**	30 (35.3)	18 (30.5)	9 (40.9)	3 (75.0)

Abbreviations: CD, Crohn’s disease; IBD, inflammatory bowel disease; IBD-U, inflammatory bowel disease-unclassified; UC, ulcerative colitis.

a The estimated population of this age group during this time was 1 043 060.^13^

The prospectively calculated incidence of pediatric IBD in New Zealand in 2025 was 8.0 per 100 000 person-years. The incidence of CD, UC and IBD-U was 5.6, 2.0 and 0.4 per 100 000 person-years respectively.

#### Demographics of pediatric IBD in New Zealand diagnosed in 2025

Of the 83 patients in 2025, 30 were female. This gave a male to female ratio of 1.8:1. The male to female ratio for the CD cohort was 2.2:1 and for the UC cohort was 1.3:1.

The median age at diagnosis for pediatric IBD in New Zealand in 2025 was 12.0 years with an IQR from 10.0 to 14.0 years. The actual range was from 4.3 to 15.9 years. IBD-U was diagnosed at younger ages (median 7.5 years) than CD and UC (median 12.2 and 12.8, respectively) (Table 1).

Of the 83 children diagnosed in 2025, 62 (74.7%) identified as European, 11 (13.3%) Indian, 5 (6.0%) Asian, 4 (4.8%) Māori, 4 (4.8%) Middle Eastern/Latin American/African and 1 (1.2%) identified as Pacific peoples. Four children identified as more than 1 ethnicity.

#### Number of children diagnosed with pediatric IBD by region within New Zealand in 2025

The regions with the highest numbers of children newly diagnosed with IBD in New Zealand in 2025, were Auckland and Canterbury with 29 (35.0%) and 20 (24.1%) patients, respectively. Three regions had no patients diagnosed with pediatric IBD in 2025, namely Northland, Tairāwhiti, and West Coast (Table 2).

**Table 2:** Number of patients diagnosed with pediatric IBD in New Zealand in 2025 per region.

Region	Incidence cohort in 2025
	*n* (%)
***n***	83
** Northland**	0
** Auckland**	29 (35.0)
** Waikato**	3 (3.6)
** Bay of Plenty**	9 (10.8)
** Tairāwhiti**	0
** Hawke’s Bay**	2 (2.4)
** Taranaki**	2 (2.4)
** Manawatū-Whanganui**	3 (3.6)
** Wellington**	8 (9.6)
** Tasman**	0
** Nelson**	1 (1.2)
** Marlborough**	1 (1.2)
** West Coast**	0
** Canterbury**	20 (24.1)
** Otago**	3 (3.6)
** Southland**	2 (2.4)

#### Disease location and extra-intestinal manifestations at diagnosis of pediatric IBD diagnosed in 2025

Data were available for disease location for all but 1 of the 83 children. Disease location at diagnosis for children diagnosed with IBD in New Zealand in 2025 is presented in Table 3.

**Table 3:** Disease location of newly diagnosed inflammatory bowel disease (IBD) in children aged 0-15 inclusive in New Zealand in 2025 (*n* = 83).

Disease location^a^	*n* with data	*n* (%) or mean (SD)
	82	
** Upper GI**		26 (31.7)
** Mid small bowel**		6 (7.3)
** Ileal**		41 (50.0)
** Colonic**		68 (82.9)
** Perianal**		12 (14.6)

a Multiple options possible so percentages may not add to 100.

Of the 83 patients diagnosed in New Zealand in 2025, 12 (14.4%) had extra-intestinal manifestations at diagnosis. Of these 12 patients, 3 (3.6%) had rheumatological involvement, 7 (8.4%) had hepatobiliary involvement and 2 (2.4%) had orofacial granulomatosis.

#### Length of symptoms prior to diagnosis

Data on the duration of symptoms was available for all but 1 of the 83 children. Of these 82 children diagnosed with pediatric IBD in New Zealand in 2025, 33 (40.2%) children had symptoms for less than 3 months prior to diagnosis, 17 (20.7%) had symptoms for 3-6 months and 32 (39.0%) had symptoms for more than 6 months.

### Prevalence cohort

#### Point prevalence of pediatric IBD in New Zealand on June 30, 2025

On June 30, 2025, there were 344 children aged less than 16 years living with IBD in New Zealand. Of these, 226 (65.7%) had CD, 85 (24.7%) had UC and 33 (9.6%) had IBD-U (Table 4). There was a change in phenotype in 33 (9.6%) of children.

**Table 4:** Prevalence, age, and sex of children aged 0-15 years inclusive with IBD in children in New Zealand and by IBD subtype on June 30, 2025.

Characteristic	IBD	CD	UC	IBD-U
***n***	344	226	85	33
**Prevalence in 2025, per 100** **000^a^**	33.0	21.7	8.1	3.2
**Age at diagnosis, median (IQR) years**	10.0 (7.7-11.8)	10.1 (8-12.1)	10.0 (7.3-11.8)	9.3 (7.2-10.7)
**Age on June 30, 2025, median (IQR) years**	13.5 (11.6-14.8)	13.6 (12.1-14.8)	13.3 (11.5-15)	12.4 (11.2-14.6)
**Sex, *n* (%)**				
** Male**	194 (56.4)	134 (59.3)	40 (47.1)	20 (60.6)
** Female**	150 (43.6)	92 (40.7)	45 (52.9)	13 (39.4)

Abbreviations: CD, Crohn’s disease; IBD, inflammatory bowel disease; IBD-U, inflammatory bowel disease-unclassified; UC, ulcerative colitis.

a The estimated population of this age group at this time was 1 043 060.^13^

The point prevalence of pediatric IBD on June 30, 2025 was 33.0 per 100 000 persons. The point prevalence for CD, UC, and IBD-U was 21.7, 8.1, and 3.2 per 100 000 persons respectively.

The point prevalence in the South Island on June 30, 2025 was 55.6/100 000 persons whereas the point prevalence in the North Island was 26.8/100 000 persons under 16 years of age (Figure 1).

**Figure 1:**
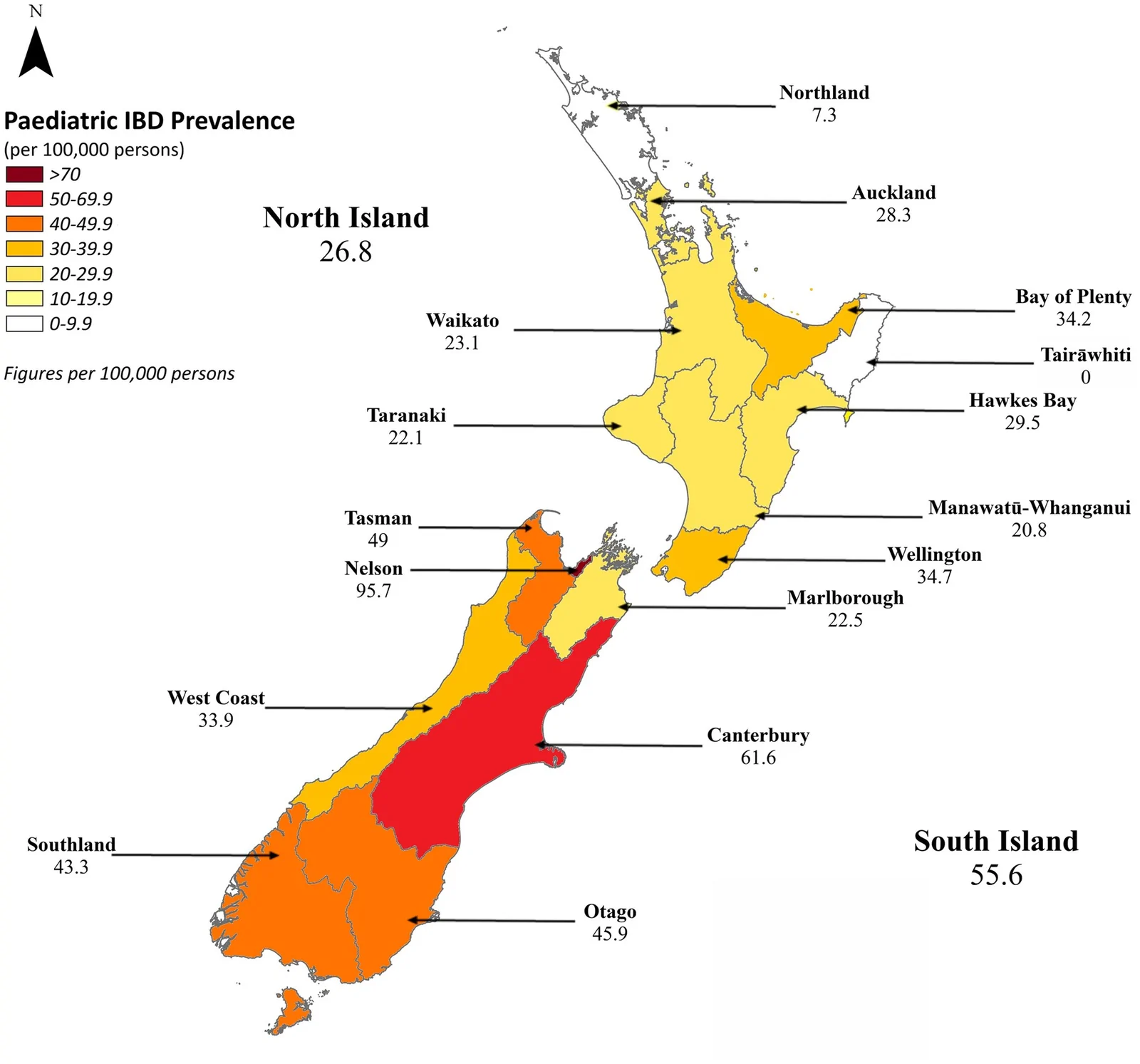
Heat map of the point prevalence by region and island of pediatric inflammatory bowel disease in New Zealand on June 30, 2025. Regional boundaries defined by Stats NZ.^14^

**Figure 2:**
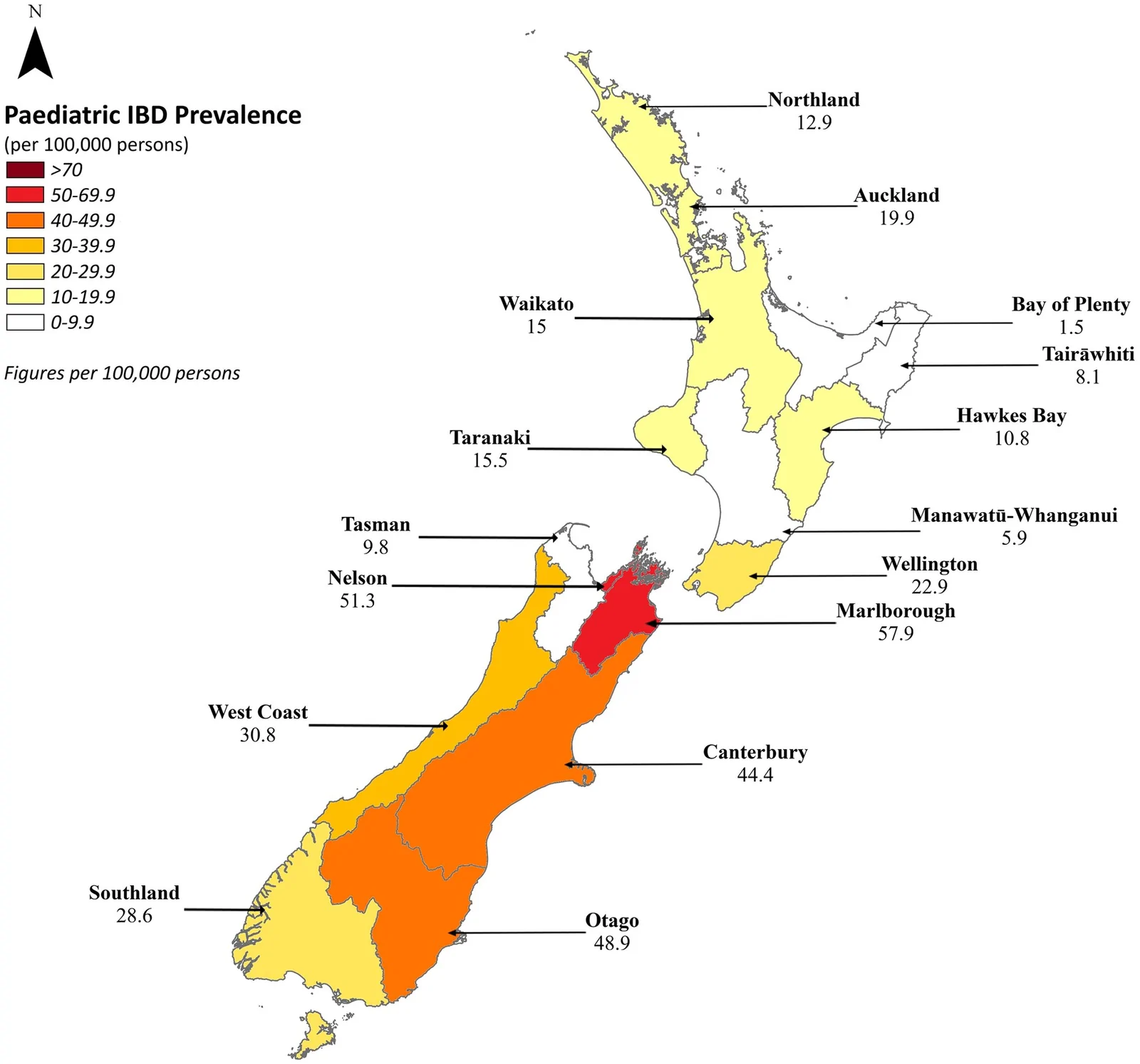
Heat map of the point prevalence by region of pediatric inflammatory bowel disease in New Zealand on June 30, 2015. Regional boundaries defined by Stats NZ.^40^

#### Demographics of pediatric IBD in New Zealand on June 30, 2025

Of the 344 patients in New Zealand on June 30, 2025, 150 (43.6%) were female. The median age of children with IBD in New Zealand on June 30, 2025 was 13.5 (IQR: 11.6-14.8) (Table 4).

Of the 344 patients, 257 (74.7%) identified as European, 52 (15.1%) Indian, 25 (7.3%) Māori, 17 (4.9%) Asian, 10 (2.9%) Middle Eastern/Latin American/African, 6 (1.7%) Pacific peoples, and 1 with another ethnicity not listed. Twenty patients identified with 2 ethnic groups, and 2 patients identified with 3 ethnic groups.

#### Key aspects of the management of pediatric IBD in New Zealand on June 30, 2025

Biologics or alternate advanced therapies were used as induction therapy at diagnosis for 19 (5.5%) of the 344 children with pediatric IBD in New Zealand on June 30, 2025. This may have been alongside other induction therapies as multiple options were available. The advanced therapies used at induction consisted of infliximab, adalimumab, and upadacitinib. The data on other induction therapies such as exclusive enteral nutrition is planned to be reported separately.

Within the prevalence cohort of 344 children, 187 (54.4%) had required escalation from a standard therapy to an advanced therapy. The median (IQR) follow-up period that is, time from diagnosis of all children with IBD to June 30, 2025, was 2.6 years (1.1-4.8 years).

Of the 344 patients, 55 (16.0%) required surgery related to their IBD. Data was not collected on the type of surgery performed or number of surgeries per patient.

The number of children with 1 or 2 admissions since diagnosis were 107 (31.1%), 33 (9.6%) had 3 or 4 admissions since diagnosis and 29 (8.4%) had 5 or more admissions since diagnosis. The maximum number of admissions since diagnosis was 22.

Two-thirds, 67.7%, (*n* = 233) had at least one of: escalation to biologic; or surgery; or an admission since diagnosis, indicating an overall pattern of severe disease, with *n* = 43 (12.5%) having all 3.

## Discussion

The incidence of pediatric IBD in New Zealand in 2025 was 8.0 cases per 100 000 person-years. The incidence of CD, UC, and IBD-U was 5.6, 2.0, and 0.4 per 100 000 person-years, respectively. The prevalence of pediatric IBD in New Zealand on June 30, 2025 was 33.0 cases per 100 000 persons, with the prevalence of CD, UC, and IBD-U being, 21.7, 8.1, and 3.2 per 100 000 persons respectively. Compared to 2015, these rates have increased from an incidence of 5.2/100 000 person-years and a prevalence of 21.7/100 000 persons.^8^^,^^9^ This shows a 53% increase in incidence, and 52% increase in prevalence (Figure 2), in pediatric IBD in New Zealand since 2015.

This increase in incidence of pediatric IBD has been seen in other parts of the world. One systematic review and meta-analysis revealed 84% of studies showed an increasing incidence of pediatric IBD, with the incidence of pediatric IBD between 2010 and 2020 highest in Canada and Northern Europe with rates of 6.4-15.4 per 100 000 person-years and 0-23.1 per 100 000 person-years respectively.^3^ Regionally, a recent meta-analysis showed a pooled annual incidence for pediatric IBD of 4.1 per 100 000 person-years between 1950 to 2020 in Australia and New Zealand—acknowledging a lack of data from other countries within Oceania.^5^ This study demonstrates the incidence of pediatric IBD in New Zealand is the highest in Australasia.^5^

This study also shows the prevalence of pediatric IBD has increased in New Zealand. In the recent meta-analysis in Oceania, the pooled prevalence rate was 36.0 per 100 000 persons–comparable to the present study.^5^ This would mean pediatric IBD in New Zealand is at Stage 2, the acceleration phase, of the epidemiological model proposed for the progression of IBD.^6^ This is consistent with most other countries which are in the emergence or acceleration phase for pediatric IBD.^3^

In this study, the incidence and prevalence of CD was higher than both UC and IBD-U. This is consistent with previous research.^3^^,^^5^^,^^8^^,^^9^ This study also shows the incidence and prevalence of pediatric IBD is higher in males than females which is consistent with prior research.^5^

In this study, 14.0% of children diagnosed with IBD in 2025, had extra-intestinal manifestations, which is comparable to the 2015 PINZ study in which 15.7% were found to have extra-intestinal manifestations at diagnosis.^9^ However, in this study fewer of these were in children diagnosed with CD (12.0% compared to 23.5% in 2015).^9^ Other studies have found the prevalence of extra-intestinal manifestations at diagnosis of pediatric IBD to be 12%.^15^

This study reported that 16.0% of all children with IBD in New Zealand in 2025, required surgery related to IBD which is comparable to the 14.6% of children who required surgery related to IBD in the 2015 PINZ study.^8^ A study in Canada found that 11.9% of children with CD had a colectomy or intestinal resection within 5 years of diagnosis and 10.9% of children with UC required a colectomy within 5 years of diagnosis.^16^ Further research into the types of surgery performed and if a change in treatment approach, for instance with upfront ileocecal resection,^17^ will be important to monitor over time.

In the 2023 census data, 62.1% in New Zealand identified as New Zealand European, 17.8% Māori, 11.2% Asian, 8.9% Pacific peoples, 5.8% Indian, 1.9% Middle Eastern/Latin American/African and 1.1% other ethnicity.^18^ In this study, 74.7% of patients identified as New Zealand European and 15.1% identified as Indian. This shows that pediatric IBD is over-represented among these ethnicities relative to the general population, consistent with prior research.^4^

Conversely, in this study Māori and Pacific children are under-represented among IBD diagnoses in New Zealand. While this is in keeping with prior research in New Zealand,^19^ and rates among indigenous populations in Australia and Canada,^5^^,^^20^^,^^21^ the number of patients identifying as Māori and Pacific in the present study is numerically higher than in the 2015 PINZ study.^8^^,^^9^ The number of patients identifying as Māori has doubled in the incidence cohort and has increased 6-fold from 4 (4.2%) to 25 (7.3%) when compared to the 2015 PINZ study in the prevalence cohort.^9^ In adult populations, the incidence of IBD has been increasing among Māori across New Zealand, with 1 study demonstrating a 5-fold increase in incidence over 20 years.^22^^,^^23^ There are known inequities for Māori in health services including, in adults, lower rates of referral to and having a colonoscopy when compared to non-Māori and lack of accessibility to healthcare services.^22^

Possible explanations for the increase in incidence of IBD among Māori include urbanization, change in diet, sanitation or other environmental changes^21^^,^^22^ and patterns of self-identification. In the prevalence cohort, 14 of the 25 patients who identified as Māori also identified as European. One study found that genetic factors may play a role, reporting lower allele frequency of CARD15 among Māori.^24^ Single nucleotide polymorphisms in the CARD15 gene have been associated with CD.^24^ Following the trend of IBD among Māori and Pacific ethnicities over time and further research into genetics among these ethnicities may uncover new genetic factors associated with protection from IBD which may help create novel gene therapies.^21^

The reasons for the overall increase in incidence of pediatric IBD are not fully understood although likely include environmental factors such as pollution, westernization of diet, urbanization of living conditions and improved disease awareness and recognition.^25^^,^^26^

The prevalence of IBD across New Zealand showed marked differences between regions with the prevalence in the South Island being 55.6 and the North Island 26.8 per 100 000 persons. This is consistent with findings from the original PINZ study and studies in both the Northern and Southern Hemispheres which show that the prevalence of IBD increases with increasing latitude.^9^^,^^27^^,^^28^ A postulated explanation for this is sun exposure, particularly as a correlate of vitamin D sufficiency.^28^^,^^29^ The link between vitamin D and IBD is mixed and further research into this theory is required.^28^ Within a New Zealand context, there are more European New Zealanders who live in the South Island (82.7%) compared to the North Island (63.1%) and this likely accounts for at least part, but not the whole, of the difference in disease burden across latitudes.^18^

Currently, there is only 1 pediatric gastroenterologist based in the South Island. In the North Island, there are 6 based in public practice, and 1 in full-time private practice. IBD makes up a significant portion of pediatric gastroenterology workload^25^ and, particularly given the high disease burden in New Zealand, an increase in the sub-specialist medical provision, particularly in the South Island would be welcome.

This study showed that 38.6% of children in New Zealand were diagnosed with IBD more than 6 months after symptom onset, comparable to rates in other parts of the world such as Canada, in which 39% of children also took more than 6 months to be diagnosed.^30^ Studies from Europe have found a median time from symptom onset to diagnosis of 4-5 months.^31^^,^^32^ Diagnostic delay of IBD in children is associated with adverse outcomes such as growth impairment, intestinal stenosis and reduced quality of life.^30^^,^^33^^,^^34^ Early detection can enable a child to function well for example by improving school attendance.^33^ Removing barriers to healthcare, increasing disease awareness^34^ and having a user-friendly healthcare system could improve diagnostic delay.^35^

While in New Zealand exclusive enteral nutrition is the first line induction treatment for CD, in this study, 5.5% of children with IBD in New Zealand on June 30, 2025, had an advanced therapy as induction and 54.4% required escalation to a biologic or other advanced therapy as part of their ongoing treatment. A study in New Zealand which completed a 3-year follow up of patients from the 2015 PINZ study showed that 37.5% of patients were receiving a biologic at follow-up.^9^^,^^36^ In New Zealand children with IBD can be prescribed a biologic if they have fistulizing disease at any time, or if they have a high disease activity score and have had unsuccessful treatment with, or intolerable side effects to, immunomodulators and corticosteroids.^37^

A study in the South Island of New Zealand found biological drugs and associated costs are the main cost drivers in the management of pediatric IBD.^38^ However, biologics are effective at inducing and maintaining remission in children with IBD.^39^ Working out an ideal strategy with regards to the efficient use of advanced medications, which may prove expensive in the short term but more economical in the long term, will be important to achieve the 2-fold priorities of optimizing patient outcomes and being fiscally responsible in the context of increasing healthcare costs and disease burden.^40^

The current study was designed and conducted in the same fashion as the 2015 PINZ study. However, a limitation of the current study is that multiple gastroenterologists or an assigned surrogate inputted data, meaning there may be some variation in how data were recorded; in the 2015 study, all data were entered by a single researcher (R.N.L.). The surveys were designed to be simple to complete, with primarily objective answers/data sought. The main authors (C.T.L.C. and R.N.L.) were frequently contacted to provide clarification around data entry. Another limitation is that the Paris classification was not used during data collection as it was in 2015. Data were still collected on disease phenotype in a different, and arguably more clinically relevant way. A further limitation is the possibility that cases may have been missed to the adult sector despite our best efforts.

A strength of this study is that all pediatric gastroenterologists in New Zealand assisted with data collection, meaning it is likely that case acquisition was as complete as possible. Adult gastroenterologists in major centers in New Zealand were also contacted to identify further cases who were 15 years and younger. It was felt unlikely among the authors of this paper that there would be any other patients cared for elsewhere. If there were cases which were missed, this would mean the incidence and prevalence reported here will, if anything, be a slight underestimate.

In conclusion, the results of this study show an increase in both incidence and prevalence of pediatric IBD nationally. This increase was seen even among previously under-represented groups. There is a clear geographic disparity between the disease burden of pediatric IBD in New Zealand. This will require an increase in medical service provision for children in the South Island.^25^ It is hoped that the results of this study will help inform optimal healthcare provision for children and young people with IBD across New Zealand.

## Data Availability

The data underlying this article are available in the article.

## References

[1] Guan Q. A comprehensive review and update on the pathogenesis of inflammatory bowel disease. J Immunol Res. 2019;2019:7247238. 10.1155/2019/7247238 PMC6914932 31886308

[2] Ricciuto A , Mack DR, Huynh HQ, et al Diagnostic delay is associated with complicated disease and growth impairment in paediatric Crohn’s disease. J Crohn’s Colitis. 2021;15:419-431. 10.1093/ecco-jcc/jjaa197 PMC7944510 32978629

[3] Kuenzig ME , Fung S, Marderfeld L, et al Twenty-first century trends in the global epidemiology of pediatric-onset inflammatory bowel disease: systematic review. Gastroenterol. 2022;162:1147-1159.e4. 10.1053/j.gastro.2021.12.282 34995526

[4] Rajasekaran V , Evans HM, Andrews A, et al Rising incidence of inflammatory bowel disease in South Asian children in New Zealand-a retrospective population-based study. J Pediatr Gastroenterol Nutr. 2023;76:749-755. 10.1097/MPG.0000000000003735 36800276

[5] Forbes AJ , Frampton CMA, Day AS, et al Descriptive epidemiology of pediatric inflammatory bowel disease in Oceania: a systematic review and meta-analysis. J Pediatr Gastroenterol Nutr. 2023;77:512-518. 10.1097/MPG.0000000000003900 37496115

[6] Hracs L , Windsor J, Gorospe J, et al Global evolution of inflammatory bowel disease across epidemiologic stages. Nature. 2025;642:458-466. 10.1038/s41586-025-08940-0 PMC12158780 40307548

[7] Forbes A , Day A, Frampton C, et al Compounding prevalence of inflammatory bowel disease in a 2024 population-based study from Canterbury, New Zealand. J Gastroenterol Hepatol. 2025;9:e70192 10.1002/jgh3.70192 PMC12125484 40452912

[8] Lopez R , Evans H, Appleton L, et al Prevalence of paediatric inflammatory bowel disease in New Zealand in 2015: initial results from the PINZ study. Inflamm Bowel Dis. 2017;23:1418-1424. 10.1097/MIB.0000000000001138 28570430

[9] Lopez R , Evans H, Appleton L, et al Prospective incidence of paediatric inflammatory bowel disease in New Zealand in 2015: results from the paediatric inflammatory bowel disease in New Zealand (PINZ) study. J Pediatr Gastroenterol Nutr. 2018;66:e122-e126. 10.1097/MPG.0000000000001806 29077643

[10] Levine A , Koletzko S, Turner D, et al The ESPGHAN revised Porto criteria for the diagnosis of inflammatory bowel disease in children and adolescents. J Pediatr Gastroenterol Nutr. 2014;58:795-806. 10.1097/MPG.0000000000000239 24231644

[11] Public Hospitals. Ministry of Health NZ. Accessed March 10, 2026. https://www.health.govt.nz/regulation-legislation/certification-of-health-care-services/certified-providers/public-hospitals

[12] Census—Stats NZ. Govt.nz. Accessed March 25. 2026. https://www.stats.govt.nz/topics/census/

[13] Infoshare—Stats NZ. Govt.nz. 2026. Accessed February 3, 2026. https://infoshare.stats.govt.nz/infoshare/ViewTable.aspx?pxID=c31cdee4-4fdc-4e79-b551-448f2b4eabce

[14] Stats NZ Geographic Data Service. Govt.nz. Stats NZ Geographic Data Service; 2026. Accessed April 17, 2026. https://datafinder.stats.govt.nz/layer/120945-regional-council-2025-clipped/.

[15] Chaparro M , Garre A, Ricart E, et al Differences between childhood- and adulthood-onset inflammatory bowel disease: the CAROUSEL study from GETECCU. Aliment Pharmacol Ther. 2019;49:419-428. 10.1111/apt.15114 30637837

[16] Kuenzig ME , Bitton A, Carroll MW, et al Health services utilization and specialist care in pediatric inflammatory bowel disease: a multiprovince population-based cohort study. Inflamm Bowel Dis. 2024;30:2356-2369. 10.1093/ibd/izae010 PMC11630251 38366807

[17] Ponsioen CY , de Groof EJ, Eshuis EJ, et al Laparoscopic ileocaecal resection versus infliximab for terminal ileitis in Crohn’s disease: a randomised controlled, open-label, multicentre trial. Lancet Gastroenterol Hepatol. 2017;2:785-792. 10.1016/S2468-1253(17)30248-0 28838644

[18] Stats NZ. Census Results Reflect Aotearoa New Zealand’s Diversity—Stats NZ. Govt.nz. Accessed May 19, 2026. https://www.stats.govt.nz/news/census-results-reflect-aotearoa-new-zealands-diversity/

[19] Rajasekaran V , Evans HM, de Lore D, et al Letter to the editor. J Paediatr Child Health. 2024;60:267-267. 10.1111/jpc.16587 38847121

[20] Leach ST , Day AS, Moore D, et al Low rate of inflammatory bowel disease in the Australian indigenous paediatric population. J Paediatr Child Health. 2014;50:328-329. 10.1111/jpc.12535 24698068

[21] Munir O , Condray C, Souza Lira AP, et al Inflammatory bowel disease in indigenous populations: a scoping review. Crohn’s Colitis 360. 2025;7:otaf057 10.1093/crocol/otaf057 PMC12578509 41179508

[22] Qiu M , Patel RN, Kerrison C, et al Low but increasing rates of inflammatory bowel disease in Māori: a report from Lakes district health board IBD. N Z Med J. 2022;135:99-105. 35728240

[23] Law D , Patel R, Gearry R, et al Phenotypes of inflammatory bowel disease in the Māori population of New Zealand. Intern Med J. 2024;54:1541-1547. 10.1111/imj.16416 38982857

[24] Gearry RB , Lea RA, Roberts RL, et al CARD15 allele frequency differences in New Zealand Maori: ancestry specific susceptibility to Crohn’s disease in New Zealand? Gut. 2006;55:580. 10.1136/gut.2005.085464 PMC1856176 16531539

[25] Ashton J , Beattie R. Inflammatory bowel disease: recent developments. Arch Dis Child. 2024;109:370-376. 10.1136/archdischild-2023-325668 PMC11056939 37468139

[26] Suarez RG , Osornio-Vargas AR, Wine E. Ambient air pollution and pediatric inflammatory bowel diseases: an updated scoping review. Dig Dis Sci. 2022;67:4342-4354. 10.1007/s10620-022-07597-3 35751831

[27] Borowitz SM. The epidemiology of inflammatory bowel disease: clues to pathogenesis? Front Pediatr. 2022;10:1103713. 10.3389/fped.2022.1103713 PMC9886670 36733765

[28] Holmes EA , Rodney Harris RM, Lucas RM. Low sun exposure and vitamin D deficiency as risk factors for inflammatory bowel disease, with a focus on childhood onset. Photochem Photobiol. 2019;95:105-118. 10.1111/php.13007 30155900

[29] Lucas RM , Valery P, van der Mei I, et al Sun exposure over a lifetime in Australian adults from latitudinally diverse regions. Photochem Photobiol. 2013;89:737-744. 10.1111/php.12044 23311500

[30] Ricciuto A , Fish JR, Tomalty DE, et al Diagnostic delay in Canadian children with inflammatory bowel disease is more common in Crohn’s disease and associated with decreased height. Arch Dis Child. 2018;103:319-326. 10.1136/archdischild-2017-313060 28794097

[31] Sawczenko A , Sandhu B. Presenting features of inflammatory bowel disease in great Britain and Ireland. Arch Dis Child. 2003;88:995-1000. 10.1136/adc.88.11.995 PMC1719349 14612366

[32] Timmer A , Behrens R, Buderus S, et al Childhood onset inflammatory bowel disease: predictors of delayed diagnosis from the CEDATA German-language pediatric inflammatory bowel disease registry. J Pediatr. 2011;158:467-473.e2. 10.1016/j.jpeds.2010.09.014 21051046

[33] Ashton JJ , Harden A, Beattie RM. Paediatric inflammatory bowel disease: improving early diagnosis. Arch Dis Child. 2018;103:307-308. 10.1136/archdischild-2017-313955 29175974

[34] Vernon-Roberts A , Day AS. Promoting early testing and appropriate referral to reduce diagnostic delay for children with suspected inflammatory bowel disease, a narrative review. Transl Pediatr. 2023;12:1416-1430. 10.21037/tp-23-35 PMC10416131 37575896

[35] Khalilipour BS , Day AS, Kenrick K, et al Diagnostic delay in paediatric inflammatory bowel disease—a systematic investigation. J Clin Med. 2022;11:4161. 10.3390/jcm11144161 PMC9316086 35887925

[36] Martin N , Roberts A, Evans H, et al Three‐year outcomes of childhood inflammatory bowel disease in New Zealand: a population‐based cohort study. JGH Open. 2020;4:642-648. 10.1002/jgh3.12310 PMC7411647 32782951

[37] Pharmac. Special Authority Forms. Accessed April 3, 2026. https://schedule.pharmac.govt.nz/SAForms.php.

[38] Park KT , Ehrlich OG, Allen JI, et al The cost of inflammatory bowel disease: an initiative from the Crohn’s & Colitis Foundation. Inflamm Bowel Dis. 2019;26:1-10. 10.1093/ibd/izz104 PMC7534391 31112238

[39] Aardoom MA , Veereman G, de Ridder L. A review on the use of anti-TNF in children and adolescents with inflammatory bowel disease. Int J Mol Sci. 2019;20:2529. 10.3390/ijms20102529 PMC6566820 31126015

[40] Zhang L , Jin Z, Hao J. Efficacy of early biologic therapy versus late/conventional therapy in children and adolescents with Crohn’s disease: a systematic review and meta-analysis. Saudi J Gastroenterol. 2023;29:259-268. 10.4103/sjg.sjg_190_23 PMC10644997 37787346

